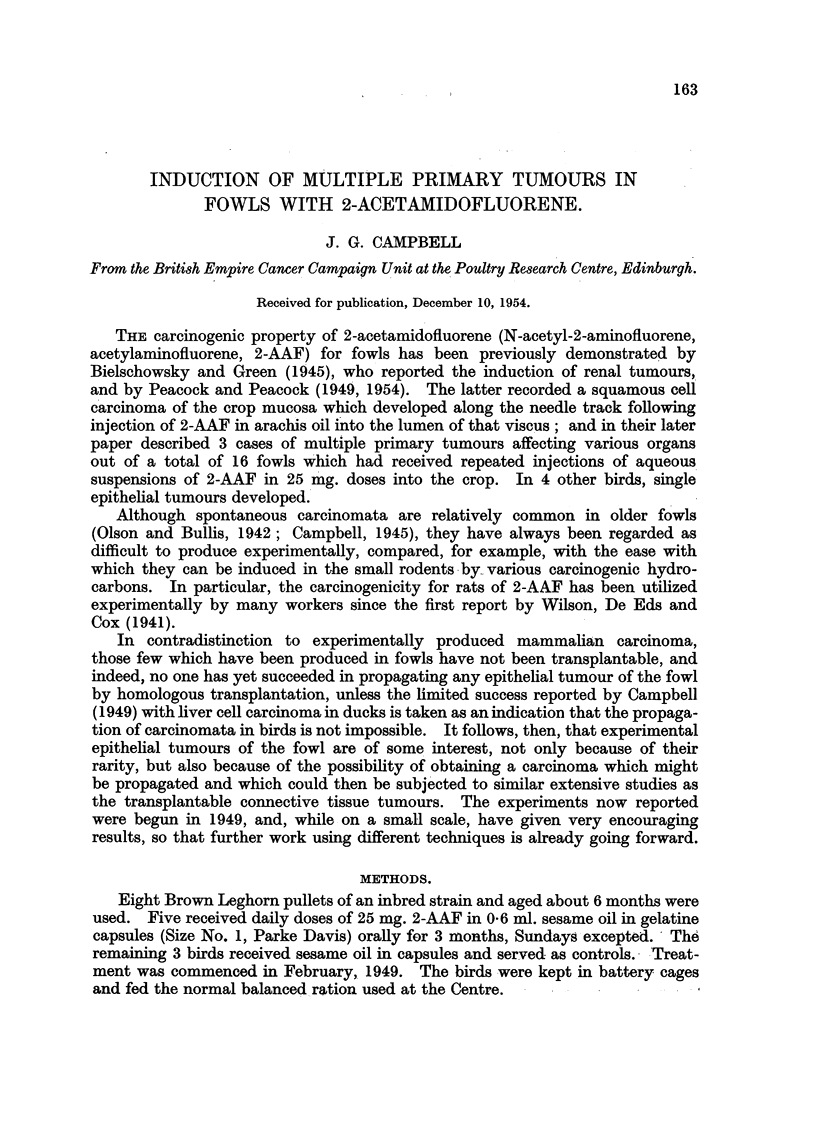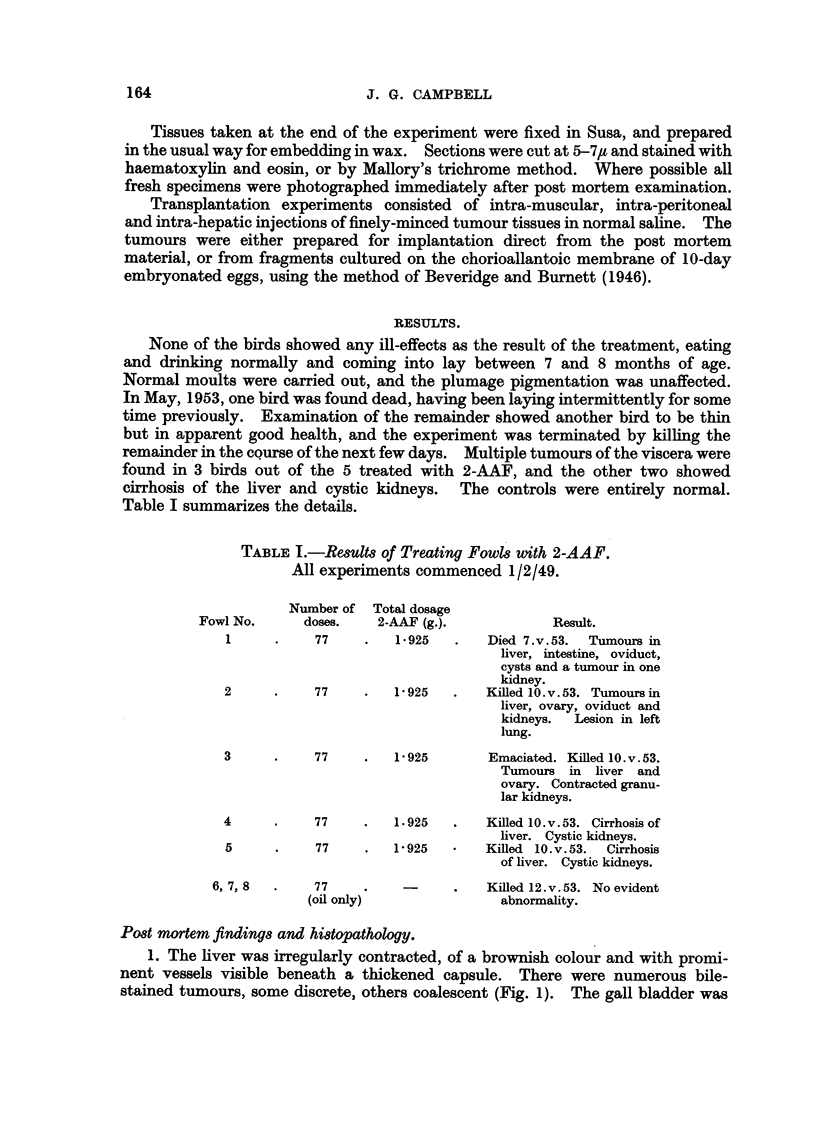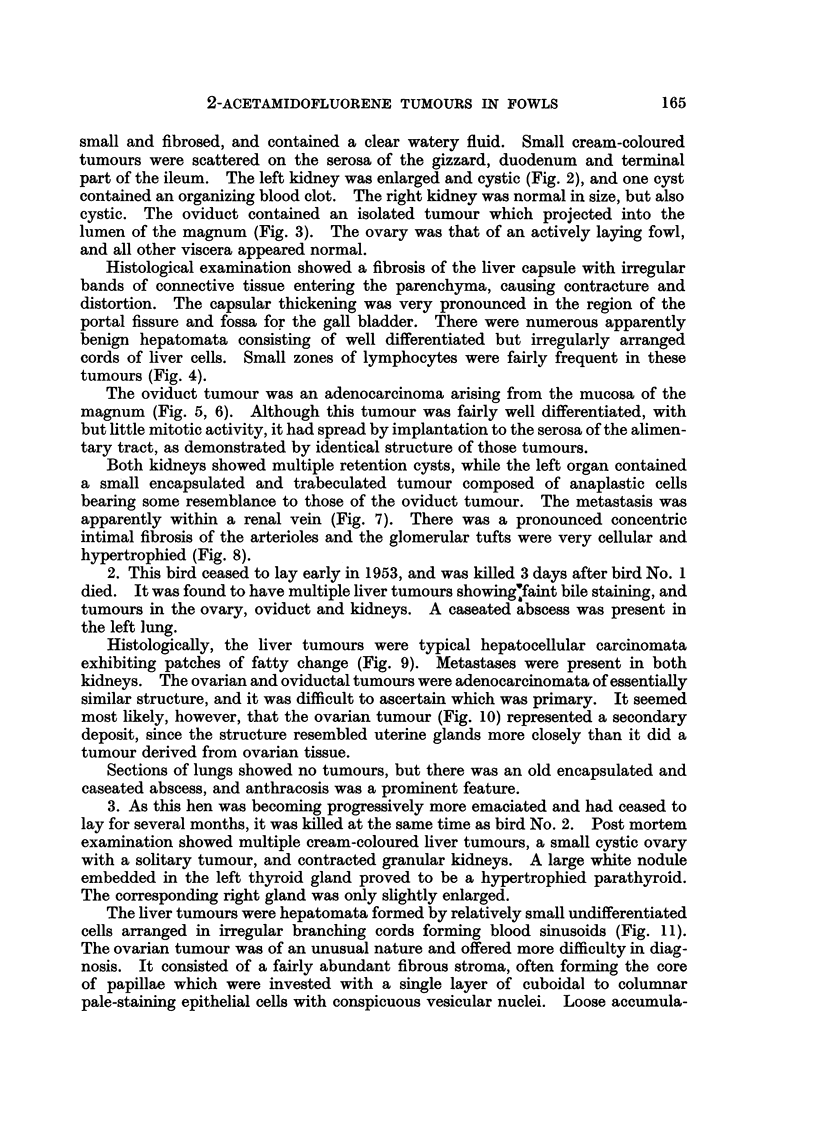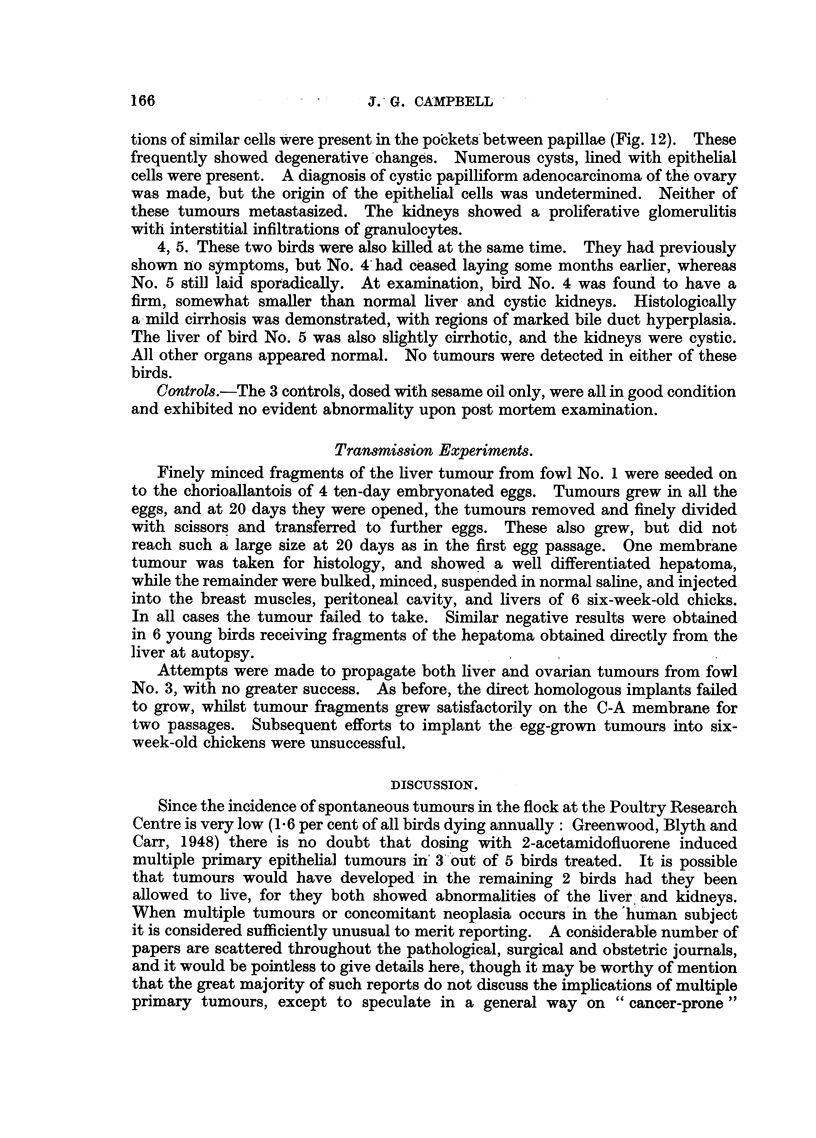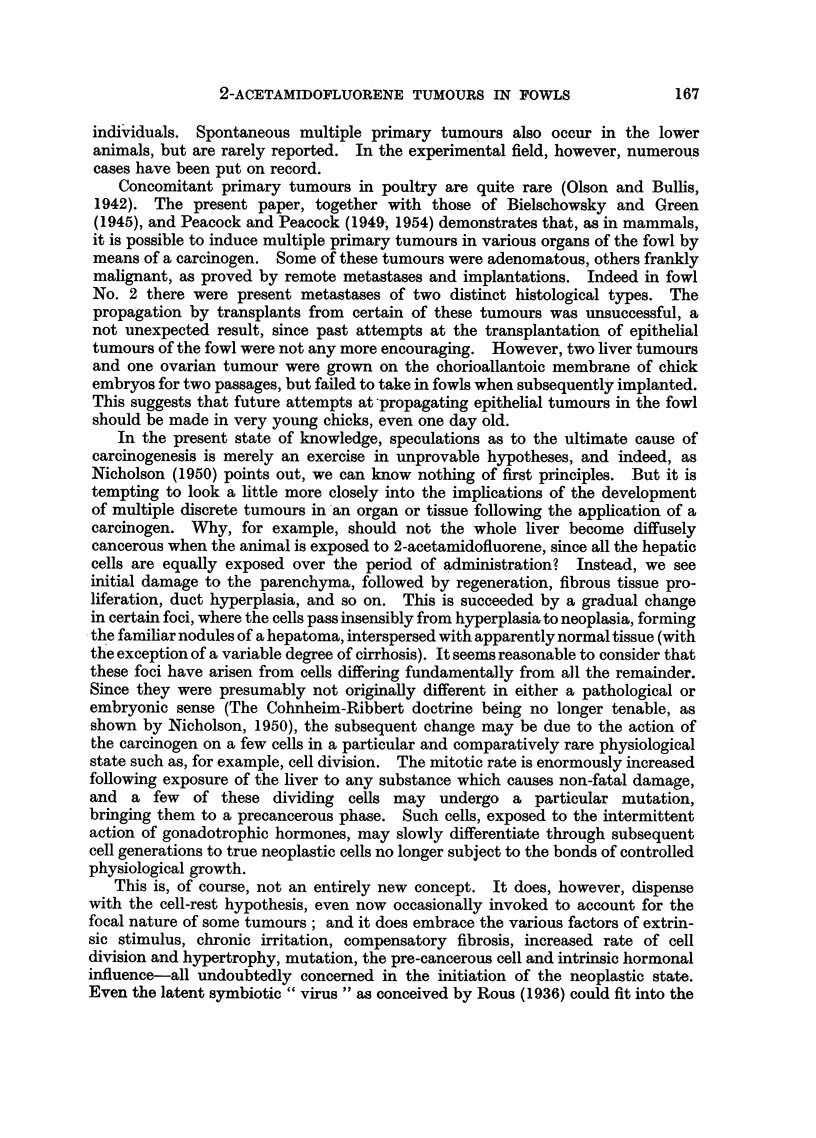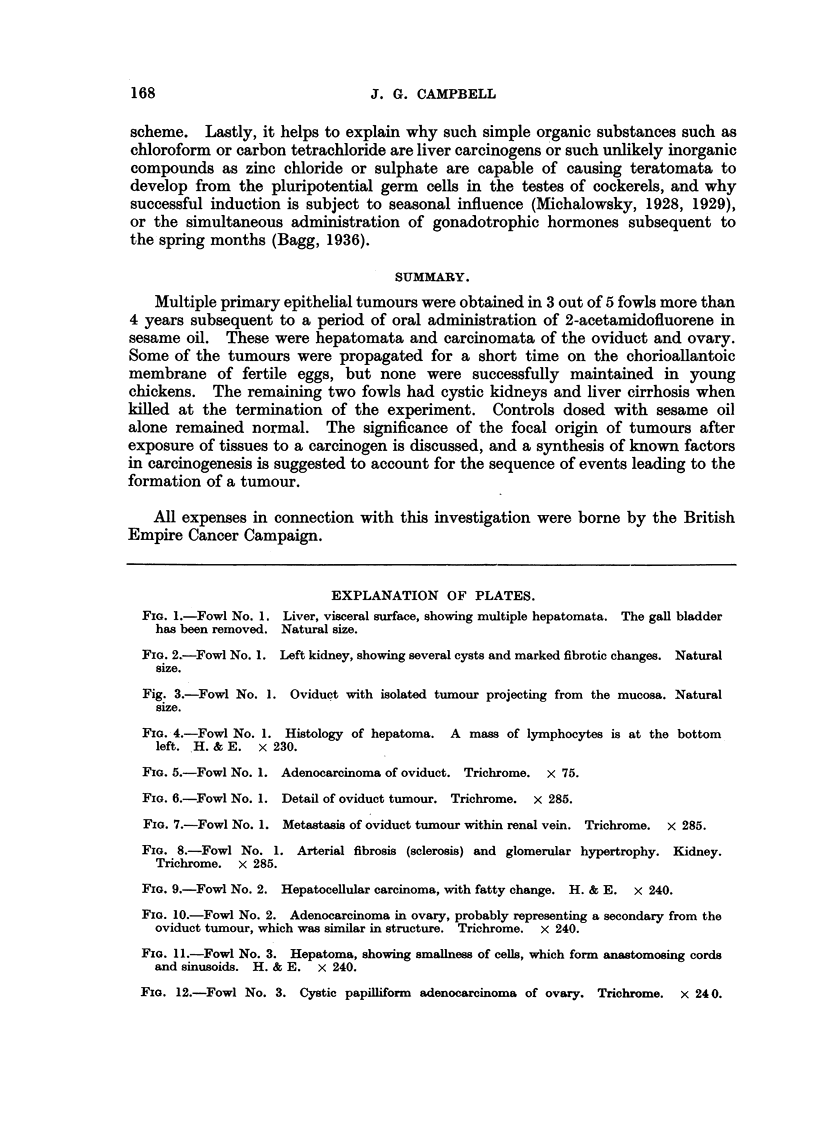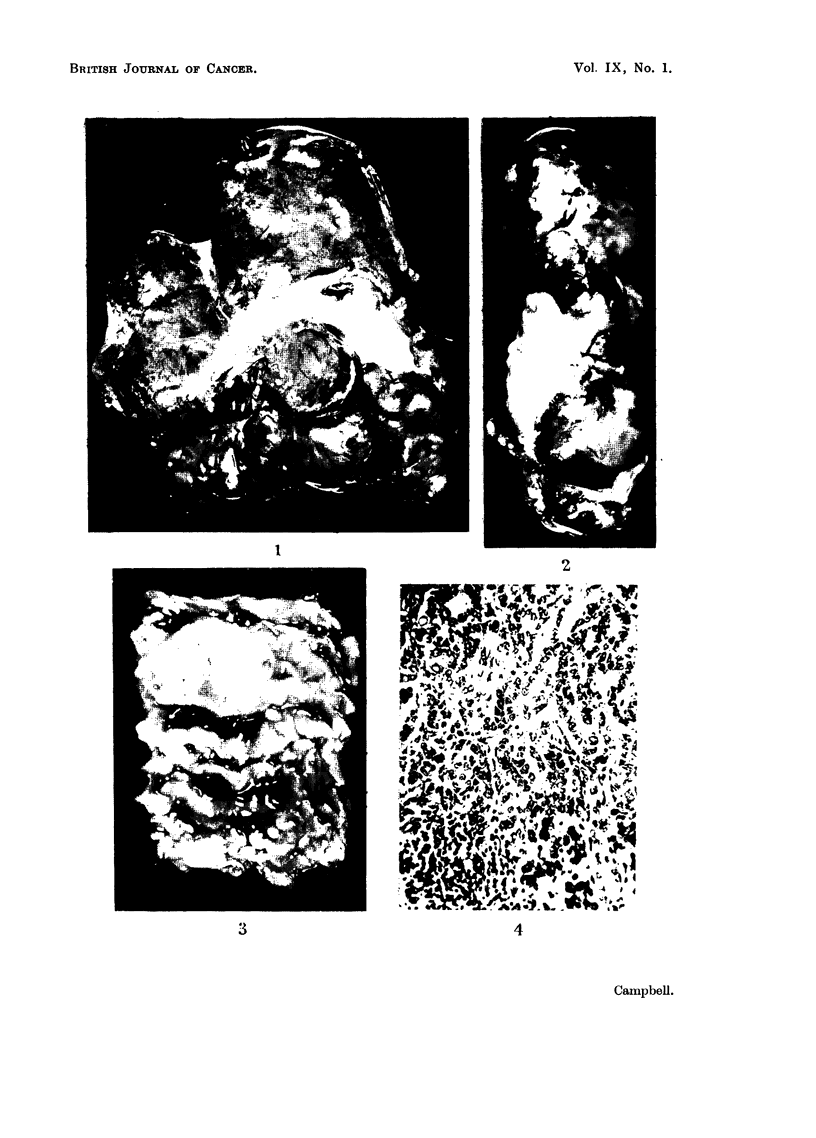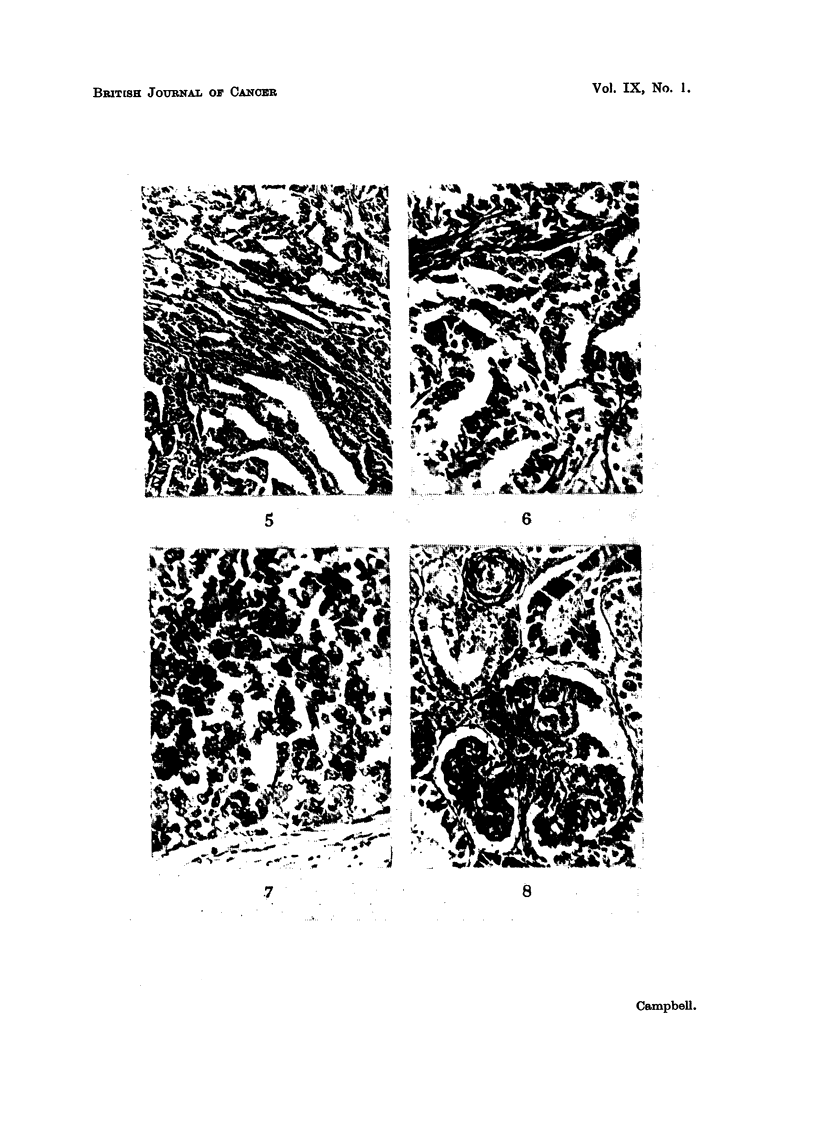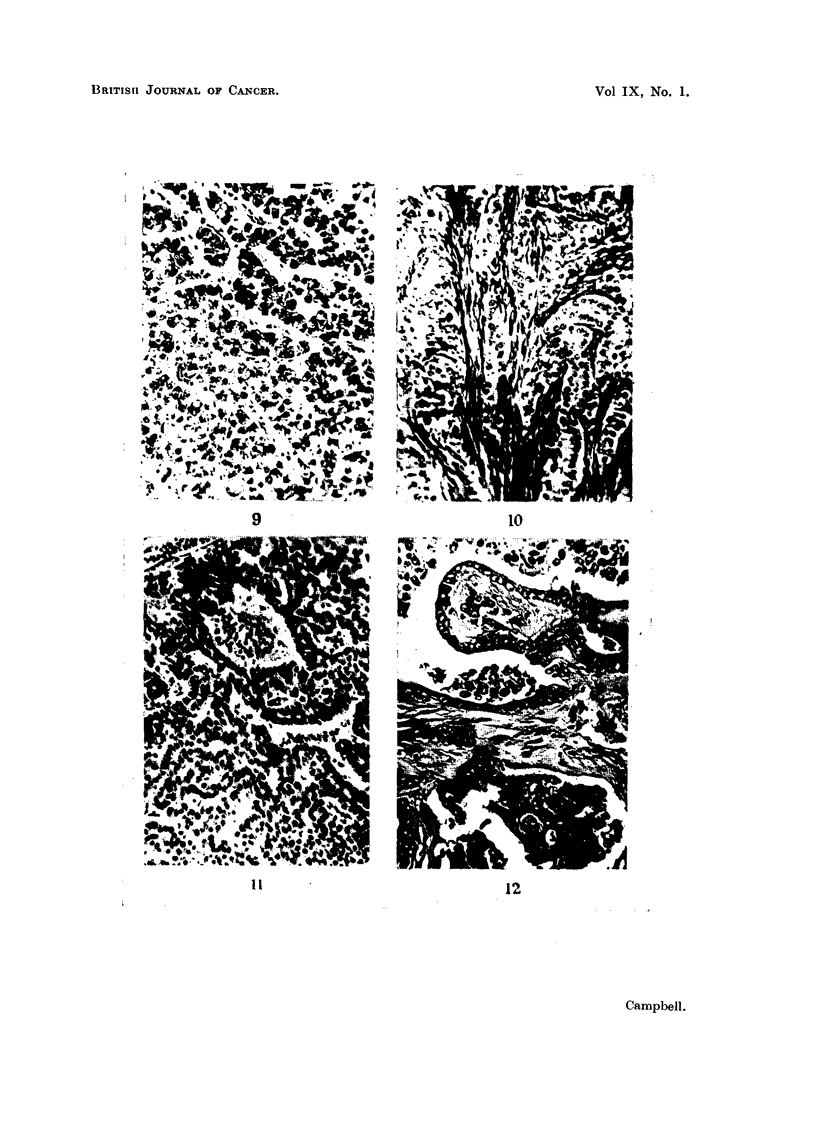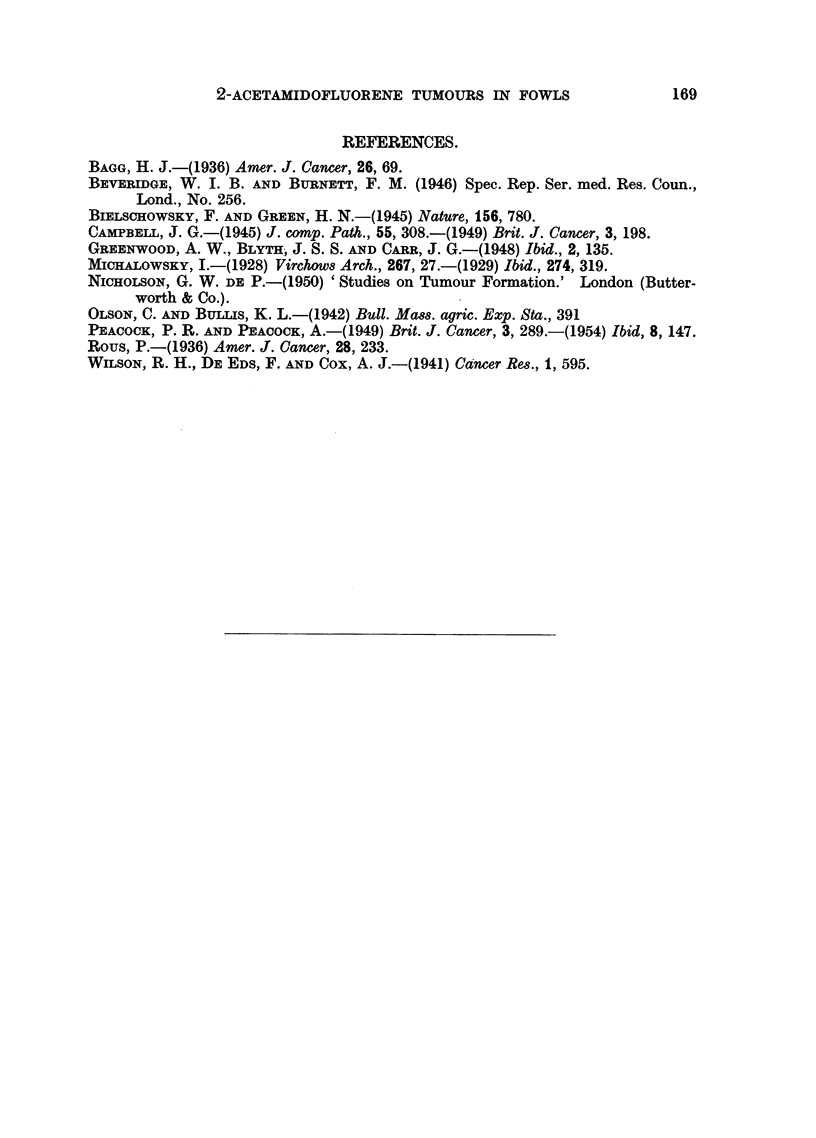# Induction of Multiple Primary Tumours in Fowls with 2-Acetamidofluorene

**DOI:** 10.1038/bjc.1955.12

**Published:** 1955-03

**Authors:** J. G. Campbell

## Abstract

**Images:**


### INDUCTION OF MULTIPLE PRIMARY TUMOURS IN FOWLS WITH 2-ACETAMIDOFLUORENE.

J. G. CAMPBELL

*From the British Empire Cancer Campaign Unit at the Poultry Research Centre, Edinburgh.*

Received for publication, December 10, 1954.

THE carcinogenic property of 2-acetamidofluorene (N-acetyl-2-aminofluorene, acetylaminofluorene, 2-AAF) for fowls has been previously demonstrated by Bielschowsky and Green (1945), who reported the induction of renal tumours, and by Peacock and Peacock (1949, 1954). The latter recorded a squamous cell carcinoma of the crop mucosa which developed along the needle track following injection of 2-AAF in arachis oil into the lumen of that viscus ; and in their later paper described 3 cases of multiple primary tumours affecting various organs out of a total of 16 fowls which had received repeated injections of aqueous suspensions of 2-AAF in 25 mg. doses into the crop. In 4 other birds, single epithelial tumours developed.

Although spontaneous carcinomata are relatively common in older fowls (Olson and Bullis, 1942 ; Campbell, 1945), they have always been regarded as difficult to produce experimentally, compared, for example, with the ease with which they can be induced in the small rodents by various carcinogenic hydrocarbons. In particular, the carcinogenicity for rats of 2-AAF has been utilized experimentally by many workers since the first report by Wilson, De Eds and Cox (1941).

In contradistinction to experimentally produced mammalian carcinoma, those few which have been produced in fowls have not been transplantable, and indeed, no one has yet succeeded in propagating any epithelial tumour of the fowl by homologous transplantation, unless the limited success reported by Campbell (1949) with liver cell carcinoma in ducks is taken as an indication that the propagation of carcinomata in birds is not impossible. It follows, then, that experimental epithelial tumours of the fowl are of some interest, not only because of their rarity, but also because of the possibility of obtaining a carcinoma which might be propagated and which could then be subjected to similar extensive studies as the transplantable connective tissue tumours. The experiments now reported were begun in 1949, and, while on a small scale, have given very encouraging results, so that further work using different techniques is already going forward.

### METHODS.

Eight Brown Leghorn pullets of an inbred strain and aged about 6 months were used. Five received daily doses of 25 mg. 2-AAF in 0·6 ml. sesame oil in gelatine capsules (Size No. 1, Parke Davis) orally for 3 months, Sundays excepted. The remaining 3 birds received sesame oil in capsules and served as controls. Treatment was commenced in February, 1949. The birds were kept in battery cages and fed the normal balanced ration used at the Centre.

Tissues taken at the end of the experiment were fixed in Susa, and prepared in the usual way for embedding in wax. Sections were cut at 5–7$\mu$ and stained with haematoxylin and eosin, or by Mallory's trichrome method. Where possible all fresh specimens were photographed immediately after post mortem examination.

Transplantation experiments consisted of intra-muscular, intra-peritoneal and intra-hepatic injections of finely-minced tumour tissues in normal saline. The tumours were either prepared for implantation direct from the post mortem material, or from fragments cultured on the chorioallantoic membrane of 10-day embryonated eggs, using the method of Beveridge and Burnett (1946).

### RESULTS.

None of the birds showed any ill-effects as the result of the treatment, eating and drinking normally and coming into lay between 7 and 8 months of age. Normal moults were carried out, and the plumage pigmentation was unaffected. In May, 1953, one bird was found dead, having been laying intermittently for some time previously. Examination of the remainder showed another bird to be thin but in apparent good health, and the experiment was terminated by killing the remainder in the course of the next few days. Multiple tumours of the viscera were found in 3 birds out of the 5 treated with 2-AAF, and the other two showed cirrhosis of the liver and cystic kidneys. The controls were entirely normal. Table I summarizes the details.

TABLE I.—*Results of Treating Fowls with 2-AAF.*
All experiments commenced 1/2/49.

| Fowl No. | Number of doses. | Total dosage 2-AAF (g.). | Result. |
|---|---|---|---|
| 1 | 77 | 1·925 | Died 7.v.53. Tumours in liver, intestine, oviduct, cysts and a tumour in one kidney. |
| 2 | 77 | 1·925 | Killed 10.v.53. Tumours in liver, ovary, oviduct and kidneys. Lesion in left lung. |
| 3 | 77 | 1·925 | Emaciated. Killed 10.v.53. Tumours in liver and ovary. Contracted granular kidneys. |
| 4 | 77 | 1·925 | Killed 10.v.53. Cirrhosis of liver. Cystic kidneys. |
| 5 | 77 | 1·925 | Killed 10.v.53. Cirrhosis of liver. Cystic kidneys. |
| 6, 7, 8 | 77 (oil only) | — | Killed 12.v.53. No evident abnormality. |

*Post mortem findings and histopathology.*

1. The liver was irregularly contracted, of a brownish colour and with prominent vessels visible beneath a thickened capsule. There were numerous bile-stained tumours, some discrete, others coalescent (Fig. 1). The gall bladder was small and fibrosed, and contained a clear watery fluid. Small cream-coloured tumours were scattered on the serosa of the gizzard, duodenum and terminal part of the ileum. The left kidney was enlarged and cystic (Fig. 2), and one cyst contained an organizing blood clot. The right kidney was normal in size, but also cystic. The oviduct contained an isolated tumour which projected into the lumen of the magnum (Fig. 3). The ovary was that of an actively laying fowl, and all other viscera appeared normal.

Histological examination showed a fibrosis of the liver capsule with irregular bands of connective tissue entering the parenchyma, causing contracture and distortion. The capsular thickening was very pronounced in the region of the portal fissure and fossa for the gall bladder. There were numerous apparently benign hepatomata consisting of well differentiated but irregularly arranged cords of liver cells. Small zones of lymphocytes were fairly frequent in these tumours (Fig. 4).

The oviduct tumour was an adenocarcinoma arising from the mucosa of the magnum (Fig. 5, 6). Although this tumour was fairly well differentiated, with but little mitotic activity, it had spread by implantation to the serosa of the alimentary tract, as demonstrated by identical structure of those tumours.

Both kidneys showed multiple retention cysts, while the left organ contained a small encapsulated and trabeculated tumour composed of anaplastic cells bearing some resemblance to those of the oviduct tumour. The metastasis was apparently within a renal vein (Fig. 7). There was a pronounced concentric intimal fibrosis of the arterioles and the glomerular tufts were very cellular and hypertrophied (Fig. 8).

2. This bird ceased to lay early in 1953, and was killed 3 days after bird No. 1 died. It was found to have multiple liver tumours showing faint bile staining, and tumours in the ovary, oviduct and kidneys. A caseated abscess was present in the left lung.

Histologically, the liver tumours were typical hepatocellular carcinomata exhibiting patches of fatty change (Fig. 9). Metastases were present in both kidneys. The ovarian and oviductal tumours were adenocarcinomata of essentially similar structure, and it was difficult to ascertain which was primary. It seemed most likely, however, that the ovarian tumour (Fig. 10) represented a secondary deposit, since the structure resembled uterine glands more closely than it did a tumour derived from ovarian tissue.

Sections of lungs showed no tumours, but there was an old encapsulated and caseated abscess, and anthracosis was a prominent feature.

3. As this hen was becoming progressively more emaciated and had ceased to lay for several months, it was killed at the same time as bird No. 2. Post mortem examination showed multiple cream-coloured liver tumours, a small cystic ovary with a solitary tumour, and contracted granular kidneys. A large white nodule embedded in the left thyroid gland proved to be a hypertrophied parathyroid. The corresponding right gland was only slightly enlarged.

The liver tumours were hepatomata formed by relatively small undifferentiated cells arranged in irregular branching cords forming blood sinusoids (Fig. 11). The ovarian tumour was of an unusual nature and offered more difficulty in diagnosis. It consisted of a fairly abundant fibrous stroma, often forming the core of papillae which were invested with a single layer of cuboidal to columnar pale-staining epithelial cells with conspicuous vesicular nuclei. Loose accumulations of similar cells were present in the pockets between papillae (Fig. 12). These frequently showed degenerative changes. Numerous cysts, lined with epithelial cells were present. A diagnosis of cystic papilliform adenocarcinoma of the ovary was made, but the origin of the epithelial cells was undetermined. Neither of these tumours metastasized. The kidneys showed a proliferative glomerulitis with interstitial infiltrations of granulocytes.

4, 5. These two birds were also killed at the same time. They had previously shown no symptoms, but No. 4 had ceased laying some months earlier, whereas No. 5 still laid sporadically. At examination, bird No. 4 was found to have a firm, somewhat smaller than normal liver and cystic kidneys. Histologically a mild cirrhosis was demonstrated, with regions of marked bile duct hyperplasia. The liver of bird No. 5 was also slightly cirrhotic, and the kidneys were cystic. All other organs appeared normal. No tumours were detected in either of these birds.

*Controls*.—The 3 controls, dosed with sesame oil only, were all in good condition and exhibited no evident abnormality upon post mortem examination.

### *Transmission Experiments.*

Finely minced fragments of the liver tumour from fowl No. 1 were seeded on to the chorioallantois of 4 ten-day embryonated eggs. Tumours grew in all the eggs, and at 20 days they were opened, the tumours removed and finely divided with scissors and transferred to further eggs. These also grew, but did not reach such a large size at 20 days as in the first egg passage. One membrane tumour was taken for histology, and showed a well differentiated hepatoma, while the remainder were bulked, minced, suspended in normal saline, and injected into the breast muscles, peritoneal cavity, and livers of 6 six-week-old chicks. In all cases the tumour failed to take. Similar negative results were obtained in 6 young birds receiving fragments of the hepatoma obtained directly from the liver at autopsy.

Attempts were made to propagate both liver and ovarian tumours from fowl No. 3, with no greater success. As before, the direct homologous implants failed to grow, whilst tumour fragments grew satisfactorily on the C-A membrane for two passages. Subsequent efforts to implant the egg-grown tumours into six-week-old chickens were unsuccessful.

### DISCUSSION.

Since the incidence of spontaneous tumours in the flock at the Poultry Research Centre is very low (1·6 per cent of all birds dying annually : Greenwood, Blyth and Carr, 1948) there is no doubt that dosing with 2-acetamidofluorene induced multiple primary epithelial tumours in 3 out of 5 birds treated. It is possible that tumours would have developed in the remaining 2 birds had they been allowed to live, for they both showed abnormalities of the liver and kidneys. When multiple tumours or concomitant neoplasia occurs in the human subject it is considered sufficiently unusual to merit reporting. A considerable number of papers are scattered throughout the pathological, surgical and obstetric journals, and it would be pointless to give details here, though it may be worthy of mention that the great majority of such reports do not discuss the implications of multiple primary tumours, except to speculate in a general way on " cancer-prone "

individuals. Spontaneous multiple primary tumours also occur in the lower animals, but are rarely reported. In the experimental field, however, numerous cases have been put on record.

Concomitant primary tumours in poultry are quite rare (Olson and Bullis, 1942). The present paper, together with those of Bielschowsky and Green (1945), and Peacock and Peacock (1949, 1954) demonstrates that, as in mammals, it is possible to induce multiple primary tumours in various organs of the fowl by means of a carcinogen. Some of these tumours were adenomatous, others frankly malignant, as proved by remote metastases and implantations. Indeed in fowl No. 2 there were present metastases of two distinct histological types. The propagation by transplants from certain of these tumours was unsuccessful, a not unexpected result, since past attempts at the transplantation of epithelial tumours of the fowl were not any more encouraging. However, two liver tumours and one ovarian tumour were grown on the chorioallantoic membrane of chick embryos for two passages, but failed to take in fowls when subsequently implanted. This suggests that future attempts at propagating epithelial tumours in the fowl should be made in very young chicks, even one day old.

In the present state of knowledge, speculations as to the ultimate cause of carcinogenesis is merely an exercise in unprovable hypotheses, and indeed, as Nicholson (1950) points out, we can know nothing of first principles. But it is tempting to look a little more closely into the implications of the development of multiple discrete tumours in an organ or tissue following the application of a carcinogen. Why, for example, should not the whole liver become diffusely cancerous when the animal is exposed to 2-acetamidofluorene, since all the hepatic cells are equally exposed over the period of administration? Instead, we see initial damage to the parenchyma, followed by regeneration, fibrous tissue proliferation, duct hyperplasia, and so on. This is succeeded by a gradual change in certain foci, where the cells pass insensibly from hyperplasia to neoplasia, forming the familiar nodules of a hepatoma, interspersed with apparently normal tissue (with the exception of a variable degree of cirrhosis). It seems reasonable to consider that these foci have arisen from cells differing fundamentally from all the remainder. Since they were presumably not originally different in either a pathological or embryonic sense (The Cohnheim-Ribbert doctrine being no longer tenable, as shown by Nicholson, 1950), the subsequent change may be due to the action of the carcinogen on a few cells in a particular and comparatively rare physiological state such as, for example, cell division. The mitotic rate is enormously increased following exposure of the liver to any substance which causes non-fatal damage, and a few of these dividing cells may undergo a particular mutation, bringing them to a precancerous phase. Such cells, exposed to the intermittent action of gonadotrophic hormones, may slowly differentiate through subsequent cell generations to true neoplastic cells no longer subject to the bonds of controlled physiological growth.

This is, of course, not an entirely new concept. It does, however, dispense with the cell-rest hypothesis, even now occasionally invoked to account for the focal nature of some tumours ; and it does embrace the various factors of extrinsic stimulus, chronic irritation, compensatory fibrosis, increased rate of cell division and hypertrophy, mutation, the pre-cancerous cell and intrinsic hormonal influence—all undoubtedly concerned in the initiation of the neoplastic state. Even the latent symbiotic " virus " as conceived by Rous (1936) could fit into the scheme. Lastly, it helps to explain why such simple organic substances such as chloroform or carbon tetrachloride are liver carcinogens or such unlikely inorganic compounds as zinc chloride or sulphate are capable of causing teratomata to develop from the pluripotential germ cells in the testes of cockerels, and why successful induction is subject to seasonal influence (Michalowsky, 1928, 1929), or the simultaneous administration of gonadotrophic hormones subsequent to the spring months (Bagg, 1936).

### SUMMARY.

Multiple primary epithelial tumours were obtained in 3 out of 5 fowls more than 4 years subsequent to a period of oral administration of 2-acetamidofluorene in sesame oil. These were hepatomata and carcinomata of the oviduct and ovary. Some of the tumours were propagated for a short time on the chorioallantoic membrane of fertile eggs, but none were successfully maintained in young chickens. The remaining two fowls had cystic kidneys and liver cirrhosis when killed at the termination of the experiment. Controls dosed with sesame oil alone remained normal. The significance of the focal origin of tumours after exposure of tissues to a carcinogen is discussed, and a synthesis of known factors in carcinogenesis is suggested to account for the sequence of events leading to the formation of a tumour.

All expenses in connection with this investigation were borne by the British Empire Cancer Campaign.

---

### EXPLANATION OF PLATES.

FIG. 1.—Fowl No. 1. Liver, visceral surface, showing multiple hepatomata. The gall bladder has been removed. Natural size.

FIG. 2.—Fowl No. 1. Left kidney, showing several cysts and marked fibrotic changes. Natural size.

Fig. 3.—Fowl No. 1. Oviduct with isolated tumour projecting from the mucosa. Natural size.

FIG. 4.—Fowl No. 1. Histology of hepatoma. A mass of lymphocytes is at the bottom left. H. & E. × 230.

FIG. 5.—Fowl No. 1. Adenocarcinoma of oviduct. Trichrome. × 75.

FIG. 6.—Fowl No. 1. Detail of oviduct tumour. Trichrome. × 285.

FIG. 7.—Fowl No. 1. Metastasis of oviduct tumour within renal vein. Trichrome. × 285.

FIG. 8.—Fowl No. 1. Arterial fibrosis (sclerosis) and glomerular hypertrophy. Kidney. Trichrome. × 285.

FIG. 9.—Fowl No. 2. Hepatocellular carcinoma, with fatty change. H. & E. × 240.

FIG. 10.—Fowl No. 2. Adenocarcinoma in ovary, probably representing a secondary from the oviduct tumour, which was similar in structure. Trichrome. × 240.

FIG. 11.—Fowl No. 3. Hepatoma, showing smallness of cells, which form anastomosing cords and sinusoids. H. & E. × 240.

FIG. 12.—Fowl No. 3. Cystic papilliform adenocarcinoma of ovary. Trichrome. × 240.

1

2

3

4

5

6

7

8

Campbell.

9

10

11

12

Campbell.

### REFERENCES.

BAGG, H. J.—(1936) *Amer. J. Cancer*, **26**, 69.
BEVERIDGE, W. I. B. AND BURNETT, F. M. (1946) Spec. Rep. Ser. med. Res. Coun., Lond., No. 256.
BIELSCHOWSKY, F. AND GREEN, H. N.—(1945) *Nature*, **156**, 780.
CAMPBELL, J. G.—(1945) *J. comp. Path.*, **55**, 308.—(1949) *Brit. J. Cancer*, **3**, 198.
GREENWOOD, A. W., BLYTH, J. S. S. AND CARR, J. G.—(1948) *Ibid.*, **2**, 135.
MICHALOWSKY, I.—(1928) *Virchows Arch.*, **267**, 27.—(1929) *Ibid.*, **274**, 319.
NICHOLSON, G. W. DE P.—(1950) 'Studies on Tumour Formation.' London (Butterworth & Co.).
OLSON, C. AND BULLIS, K. L.—(1942) *Bull. Mass. agric. Exp. Sta.*, 391
PEACOCK, P. R. AND PEACOCK, A.—(1949) *Brit. J. Cancer*, **3**, 289.—(1954) *Ibid*, **8**, 147.
ROUS, P.—(1936) *Amer. J. Cancer*, **28**, 233.
WILSON, R. H., DE EDS, F. AND COX, A. J.—(1941) *Cancer Res.*, **1**, 595.